# A case of secukinumab-induced psoriasis in a 25-year-old man with improved hidradenitis suppurativa

**DOI:** 10.1016/j.jdcr.2024.08.027

**Published:** 2024-09-12

**Authors:** Faraz Yousefian, Victoria Griffith, Amanda Stallings, Katherine Rupley

**Affiliations:** a Department of Dermatology and Mohs Surgery, Philadelphia College of Osteopathic Medicine, Roswell, Georgia; b Goodman Dermatology, Roswell, Georgia; c Memorial Healthcare System, Pembroke Pines, Florida

**Keywords:** biologics, hidradenitis suppurativa, psoriasis, secukinumab

## Introduction

Hidradenitis suppurativa (HS) is a chronic, inflammatory, recurrent cutaneous condition most commonly affecting intertriginous areas in both men and women.^1^ HS has been found to affect 1% to 4% of the global population, usually manifesting between the ages 18 and 29 years, and affecting women 3 times more than men.^1^ HS is derived from hair follicles and has the propensity to form nodules, abscesses, fistulas, and sinus tracts as a result of a large inflow of proinflammatory mediators including interferon gamma, tumor necrosis factor-alfa (TNF-α), interleukin 1 (IL-1), IL-17, and IL-12/23.^1^ In late 2023, secukinumab- an anti–IL-17A antibody, was approved by the Food and Drug Administration for the treatment of HS.^2^ Secukinumab has an impressive safety profile, however a unique adverse event in the form of paradoxical psoriasis (PP) should be on a clinician’s radar when utilizing this biologic.^3^

We present a case of a patient with HS treated with secukinumab. To our knowledge, this is the first reported case of secukinumab-induced inverse psoriasis in a patient with HS.

## Case presentation

**Fig 1:**
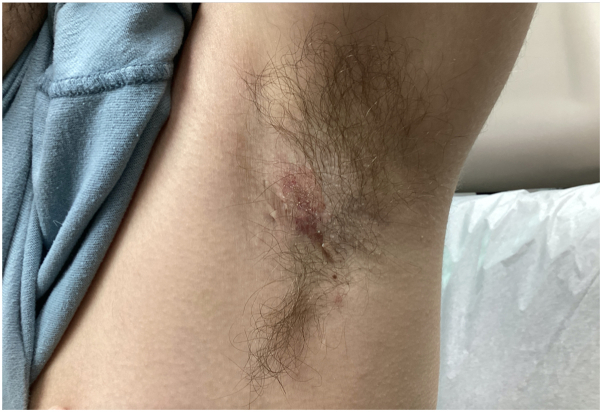
Physical examination revealing a single, tender nodule without sinus tract formation and minimal scarring, representing Hurley stage 1 disease of the left axilla.

**Fig 2:**
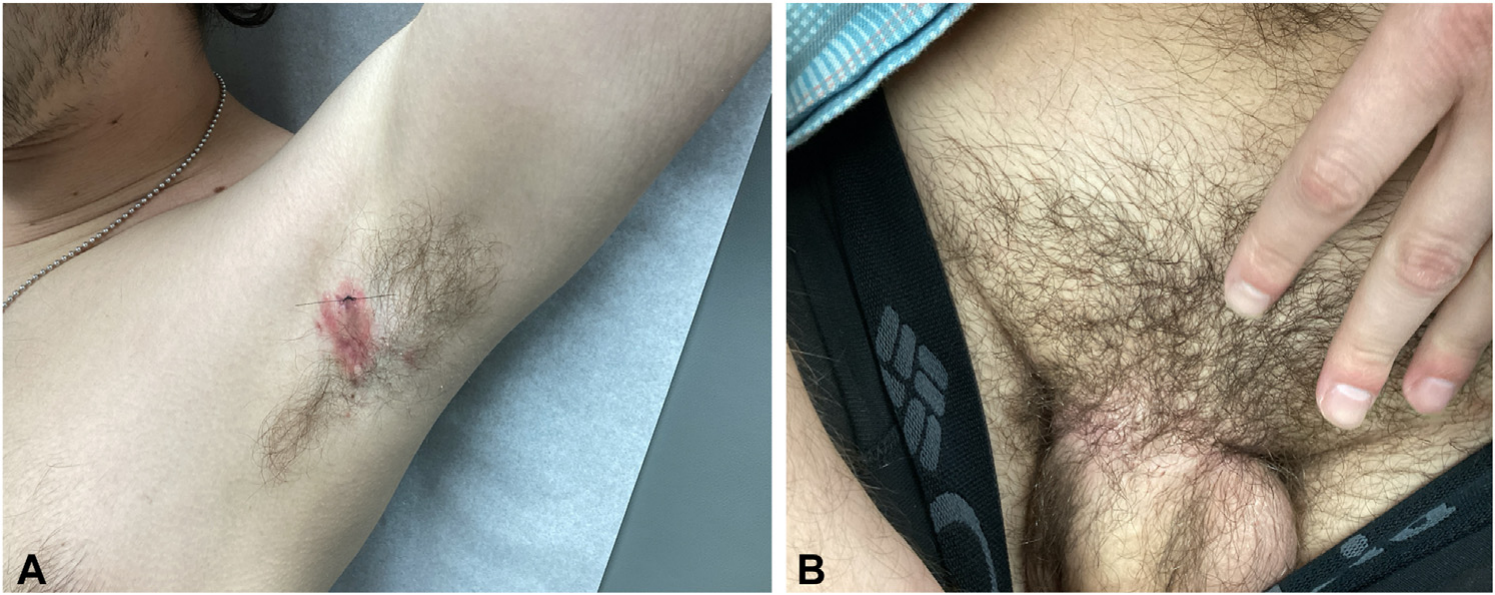
Physical examination revealing erythematous papules and annular coalescing plaques with slight crusting located on the (**A**) left axilla and (**B**) groin approximately 8 weeks into secukinumab treatment.

**Fig 3:**
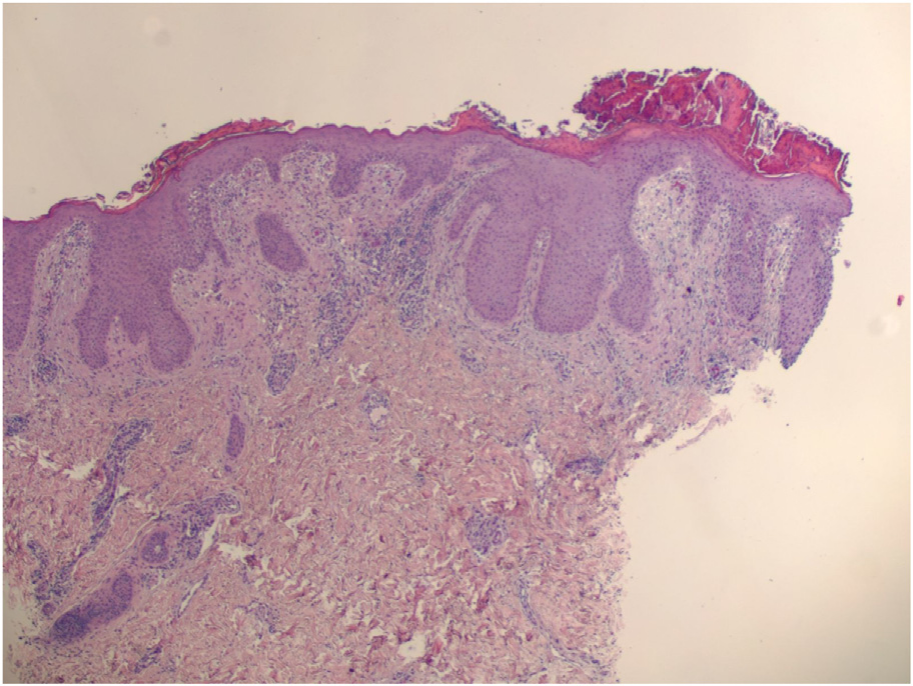
Histopathology revealing psoriasiform epidermal hyperplasia with elongated rete ridges and thinning of the suprapapillary plates. Hematoxylin and eosin stain.

A 25-year-old man with a medical history of Sjögren syndrome and HS presented with an unresolved rash on his left axilla and groin. He had previously treated his HS with doxycycline, clindamycin 1% lotion, and Hibiclens solution with minimal improvement and frequent flares. Physical examination showed a single, tender nodule without sinus tract formation and minimal scarring, representing Hurley stage 1 disease of the left axilla (Fig 1). After appropriate laboratory work and rheumatology approval, the patient was started on secukinumab 300 mg/mL subcutaneously biweekly because of its recalcitrance to prior treatment. The patient experienced rapid resolution of HS while on secukinumab and denied having any HS flares in the first 2 months of treatment. However, approximately 8 weeks into treatment he progressively developed erythematous papules and annular coalescing plaques with slight scale located on the groin, buttocks, and axilla (Fig 2). The patient denied any sick contacts, recent travel, and any medication changes. Histopathologic punch biopsy of the left axilla revealed psoriasiform epidermal hyperplasia with elongated rete ridges and thinning of the suprapapillary plates (Fig 3). The patient was diagnosed with drug-induced psoriasis and discontinuation of secukinumab resolved the recent lesions.

## Discussion

The pathogenesis of HS has previously been poorly understood, with a consensus that it is likely a multifactorial disease influenced by a genetic predisposition, inflammatory dysregulation, and modifiable environmental factors.^4^ Inflammatory dysregulation results in chronically draining fistulas in apocrine gland-bearing areas of the body, and patients are often left with extensive scarring. Treatment of HS is challenging secondary to the wide clinical manifestations and complex pathogenesis of the disease.^4^ Therapeutic options include topical therapy, systemic treatments, biologic agents, light therapy, and surgery. Treatment of choice should be selected in relation to disease severity. These options have produced variable results, and in most cases a combination of treatments is required.^4^

The knowledge of HS pathogenesis is rapidly expanding, with IL-17 cytokines emerging as key players.^2^ Secukinumab, a human monoclonal immunoglobulin G1 kappa antibody that selectively binds to IL-17A, was shown to be efficacious in both the SUNSHINE and SUNRISE randomized trials for the treatment of moderate-to-severe HS.^4^^,^^5^ These studies were identical multicenter, randomized, placebo-controlled, double blind phase 3 trials done in 219 primary sites and 40 countries that revealed secukinumab, when given every 2 weeks at a dose of 300 mg, was clinically effective at rapidly improving signs and symptoms of HS when assessed at week 16, with sustained response up to 52 weeks.^5^

Secukinumab is Food and Drug Administration-approved for the treatment of psoriasis (2015), psoriatic arthritis (2016), enthesitis-related arthritis and axial spondylitis (2016), and HS (2023). It is used off-label for rheumatoid arthritis, systemic lupus erythematosus, familial Mediterranean fever, and tumor necrosis factor receptor-associated periodic syndrome.^6^ Adverse reactions include nasopharyngitis, upper respiratory infection and diarrhea (>1%). Other adverse effects have been reported including inflammatory bowel disease and exacerbation of Crohn’s disease, various infections, some cancers, immunosuppression, and flu-like symptoms.^6^

PP is a unique type of psoriasis that may occur by exacerbating pre-existing psoriatic lesions or inducing new-onset psoriasis during treatment with a biologic drug as seen in this report.^7^ Anti–TNF-α drugs have been reported to cause PP in literature for >15 years, and cases are increasing as the use of anti–TNF-α drugs becomes more widespread.^7^ Recently, evidence of other biologics causing PP has been increasing to include anti–IL-12/23, IL-17, and IL-23 biologics.^8^ Secukinumab is among the anti–IL-17 biologics that has been reported to cause PP, although the reports are few.^3^^,^^8^

We hypothesize that the blockade of IL-17A by secukinumab may (a) cause a compensatory hyperexpression of cytokines earlier in the cascade, such as IL-12, IL-23, and TNF-α, or (b) the remaining IL-17 members (IL-B-F) may remain active, or even hyperactive, contributing to the onset of psoriatic inflammation. Further, recent studies have shown the prevalence of psoriasis in patients with HS compared with control subjects is substantially increased with a multivariate odds ratio of 4.6. It is likely that this is because of the common pathogenic pathways in which IL-17 and IL-23 play a role.^9^ It is widely recognized that IL-23 activates CD4^+^ helper T cells, which in turn release IL-17A and IL-17F. However, other cell subtypes can also produce these ILs independently of IL-23, and dysregulation in either pathway can result in excessive IL-17 production.^10^ It is possible that inhibition of IL-17A and IL-17F in a dual manner, as with bimekizumab, or direct inhibition of the IL-17A receptor itself, as with brodalumab, could provide a more efficacious outcome than IL-17A binding alone.^9^^,^^10^

It is important to note single nucleotide polymorphisms influence the transcription of TNF and other susceptibility genes related to psoriasis, possibly making PP a factor of genetic susceptibility.^7^ It is likely a combination of these factors that disrupts the complex balance of the immune system and contributes to the onset of PP. When deciding on treatment for future cases, it is important to weigh the severity of both HS and PP. In mild instances, the offending agent can be continued, with the addition of topical treatments, phototherapy, or systemic therapies. For moderate-to-severe cases, the offending agent may need to be discontinued and an alternative chosen.^8^

## Conflicts of interest

None disclosed.
